# From macrophage polarization to clinical translation: immunomodulatory hydrogels for infection-associated bone regeneration

**DOI:** 10.3389/fcell.2025.1684357

**Published:** 2025-09-24

**Authors:** Rui Zhang, Suk Fei Tan, Ye Wang, Junxue Wu, Chao Zhang

**Affiliations:** ^1^ Department of Orthopedic Surgery, Affiliated Hospital of North Sichuan Medical College, Nanchong, Sichuan, China; ^2^ School of Graduate Studies, Post Graduate Centre, Management and Science University, Shah Alam, Malaysia; ^3^ School of Pharmacy, Management and Science University, Shah Alam, Malaysia

**Keywords:** immunomodulatory hydrogels, macrophage polarization, bone regeneration, osteomyelitis, osteoimmunology, infection control, tissue engineering

## Abstract

Bone infections such as osteomyelitis and fracture-related infections are a significant clinical challenge, characterized by complex interactions between pathogenic microorganisms, disrupted immune responses, and impaired regenerative processes. A pathological hallmark of these conditions is the persistent pro-inflammatory macrophage (M1) polarization, which prevents the essential transition to anti-inflammatory M2 macrophages required for successful bone healing. This review examines the emerging paradigm of immunomodulatory hydrogels as a multifaceted therapeutic strategy that addresses both infection control and bone regeneration through targeted modulation of macrophage polarization. We systematically analyze the fundamental role of macrophage phenotypic switching in osteoimmune responses, demonstrating how infection disrupts the normal M1-to-M2 transition and perpetuates a chronic inflammatory state that impairs osteogenesis while promoting bone resorption. The review details innovative hydrogel design strategies that incorporate antimicrobial agents, immunomodulatory factors, and bioactive components to create materials capable of eliminating pathogens while simultaneously steering macrophages toward a pro-regenerative phenotype. Key approaches include integration of sequential drug-release systems, reactive oxygen species (ROS)-scavenging mechanisms, photothermal activation, and cell delivery platforms within biodegradable hydrogel matrices. Recent advances in multifunctional hydrogel systems have demonstrated superior performance compared to conventional treatments–including enhanced bacterial clearance, accelerated bone healing, and reduced infection recurrence rates in preclinical models. The pathway from laboratory findings to clinical application is critically evaluated, addressing challenges in biocompatibility, manufacturing consistency, regulatory approval, and clinical trial design. This comprehensive analysis reveals that immunomodulatory hydrogels represent a promising convergence of infection control and regenerative medicine, offering new therapeutic avenues for treating complex bone defects where traditional approaches have proven insufficient.

## 1 Introduction

Osteomyelitis and fracture-related infection (FRI) remain major clinical challenges, often leading to impaired healing and significant morbidity (Graan and Balogh, 2022; Hellwinkel et al., 2022). Despite advances in surgical debridement and antibiotic therapy, chronic bone infections have high recurrence rates (20%–30%) and can result in large defect areas with poor regenerative capacity (Qin et al., 2024). These infections are frequently caused by *Staphylococcus aureus*, which accounts for over 70% of osteomyelitis cases due to its aggressive invasion, biofilm formation, and immune evasion strategies (Nasser et al., 2020; Masters et al., 2022). The excessive release of pro-inflammatory cytokines (e.g., TNF-α, IL-1β) during chronic infection skews the bone microenvironment toward osteoclastogenesis and bone resorption while inhibiting osteogenesis, ultimately leading to bone destruction (Oliveira et al., 2020; Zhou et al., 2022). In healthy circumstances, bone repair is orchestrated by a finely tuned interplay between the skeletal and immune systems–a field known as osteoimmunology (Takayanagi and Otsuka, 2007). In the context of infection, however, this balance is lost: invading microbes trigger a sustained immune response that fails to eradicate the pathogen and simultaneously impairs regenerative processes (Qin et al., 2024).

Osteomyelitis relevant to hydrogel application spans three clinically distinct scenarios. (A) Pediatric hematogenous osteomyelitis is typically acute and mono-microbial (most often staphylococcal) in well-perfused bone and usually does not create a substantial osseous void; thus, defect-filling hydrogels are not routine, except in selected refractory cases where subperiosteal or intramedullary depots may be considered (Woods et al., 2021). (B) Post-traumatic/post-fracture osteomyelitis commonly involves hardware, biofilm, and segmental bone loss after radical debridement; here, intralesional hydrogel filling can couple high local antimicrobial levels with macrophage reprogramming and microenvironment repair—this review primarily targets scenario (B) (Metsemakers et al., 2020). (C) Chronic ischemic polymicrobial infection associated with pressure sores or diabetic-foot disease features poor perfusion with frequent anaerobes; in this setting, hydrogels are considered mainly as subperiosteal/topical adjuncts alongside revascularization and systemic therapy (Senneville et al., 2024). Because design constraints, release kinetics, and safety testing differ across scenarios, we explicitly indicate scenario applicability and delivery routes (intralesional, intramedullary, subperiosteal/topical) in subsequent sections. Staging frameworks such as Cierny–Mader help contextualize host status and anatomic involvement (Cierny et al., 2003).

Current management follows a staged, multidisciplinary pathway: radical debridement to viable, bleeding bone; deep-tissue sampling with subsequent culture-guided systemic antibiotics; dead-space management after debridement; definitive soft-tissue coverage; and stable fixation with early rehabilitation Careri et al. (2019). Pediatric acute hematogenous osteomyelitis is usually treated without defect-filling materials, whereas post-traumatic/post-fracture infection commonly leaves segmental voids requiring local reconstruction; chronic ischemic, polymicrobial disease additionally demands restoration of perfusion. Within this framework, immunomodulatory hydrogels are positioned at the defect stage as intralesional fillers that deliver high local antimicrobial levels while limiting systemic toxicity, provide host-directed cues—promoting macrophage M1→M2 transition and supporting angiogenesis/osteogenesis—and serve as a conformal, degradable scaffold for regeneration (Metsemakers et al., 2020; Senneville et al., 2024; Woods et al., 2021).

Additionally, the rise of antibiotic-resistant organisms further complicates treatment, as seen with methicillin-resistant *S. aureus* and other nosocomial pathogens that withstand conventional antibiotics (Li and Webster, 2018). These challenges have spurred interest in innovative therapeutic approaches that not only eliminate infection but also actively promote bone regeneration (Sheehy et al., 2025). One emerging strategy is to harness and modulate the body’s own immune response to create a more favorable healing environment–essentially tipping the balance from chronic inflammation to pro-regenerative immunity (Ko and Lee, 2022). In particular, macrophages have come into focus as key regulators of inflammation and tissue repair. The concept of “immune-informed regeneration” suggests that biomaterials can be engineered to interact with immune cells (such as macrophages) to encourage outcomes that aid tissue repair (Liu and Segura, 2020; Whitaker et al., 2021).

Hydrogels have gained prominence in bone tissue engineering as versatile scaffolds due to their high water content, tunable mechanical properties, and capacity to deliver cells or bioactive factors. Unlike rigid implants, hydrogels can conform to complex bone defect geometries and and can be delivered as intralesional fillers into debrided bone defects via minimally invasive approaches–an advantage in infected wounds where multiple surgeries may have compromised soft tissue (Cao et al., 2024). Early generations of hydrogels were designed to be “inert” placeholders or simple drug carriers. However, a new paradigm has emerged: immunomodulatory hydrogels that actively engage the host immune system. By incorporating immunoregulatory cues, these hydrogels aim to mitigate excessive inflammation, enhance the clearance of bacteria, and stimulate the subsequent phases of healing (He et al., 2024). In parallel, hydrogels can be formulated to release antibiotics or antimicrobial nanoparticles locally, achieving high concentrations at the infection site while sparing the patient systemic toxicity (Ahmad et al., 2024). Thus, immunomodulatory hydrogels represent a convergence of antimicrobial therapy and regenerative medicine: they seek to eradicate pathogens while concurrently guiding the immune response to facilitate bone regeneration.

This review discusses the role of macrophage polarization in bone healing and the mechanisms by which infection disrupts this critical process. It further examines recent advances in the design of immunomodulatory hydrogel systems for bone regeneration, emphasizing strategies that create a favorable immune microenvironment—particularly through targeted modulation of macrophage phenotypes—even in the presence of infection. Additionally, we explore the latest progress toward the clinical translation of these biomaterial-based approaches, summarizing encouraging outcomes from preclinical investigations as well as ongoing challenges impeding clinical implementation. By integrating osteoimmunology, materials science, and infection biology, immunomodulatory hydrogels emerge as a promising, multifaceted therapeutic strategy for treating infection-associated bone defects, with the potential to enhance clinical outcomes for patients with these challenging conditions.

## 2 Macrophage polarization and osteoimmune responses in bone regeneration

Macrophages are innate immune cells that play a pivotal role in orchestrating tissue repair, including bone healing. Upon injury or implantation of a biomaterial, circulating monocytes are recruited to the site and differentiate into tissue macrophages. These macrophages exhibit remarkable plasticity, spanning a spectrum of functional phenotypes broadly classified as pro-inflammatory “M1” or anti-inflammatory “M2” (Martin and García, 2021; Song et al., 2023). Classical M1 macrophages are activated by signals such as interferon-γ (IFN-γ) or bacterial endotoxin (LPS) and produce high levels of inflammatory cytokines (e.g., TNF-α, IL-1β, IL-6) and reactive oxygen species. M1 macrophages are effective at pathogen defense and initiating inflammation. In contrast, M2 (alternatively activated) macrophages arise in response to stimuli like IL-4, IL-13, or IL-10 and secrete anti-inflammatory cytokines (e.g., IL-10, TGF-β) along with growth factors (VEGF, PDGF, BMP-2) that support tissue repair and remodeling (Martinez et al., 2008; Liu et al., 2014). It is now appreciated that successful bone regeneration requires a carefully timed transition from an M1-dominated early response to an M2-dominated later response (Zhang D. et al., 2023). In the initial days after a fracture or bone injury, M1 macrophages help clear debris and prevent infection, producing cytokines that recruit other immune cells and osteoprogenitors to the site. Notably, M1 macrophages can enhance mesenchymal stem cell migration and osteogenesis through the COX-2/prostaglandin E2 pathway (Lu et al., 2017; McCauley et al., 2020). However, as healing progresses, a switch toward M2 macrophages is critical to resolve inflammation and stimulate new tissue formation (Schlundt et al., 2018). M2 macrophages support angiogenesis by releasing pro-angiogenic factors like VEGF, and they enhance osteogenesis by producing factors such as TGF-β1 and BMP-2 that act on mesenchymal stem cells (MSCs) and osteoblasts (Wang et al., 2017; Muñoz et al., 2020). In line with these roles, studies have shown that depleting macrophages or preventing their M1-to-M2 transition severely impairs bone healing, leading to delayed or failed fracture union (Cottrell et al., 2019; Hozain and Cottrell, 2020).

**FIGURE 1:**
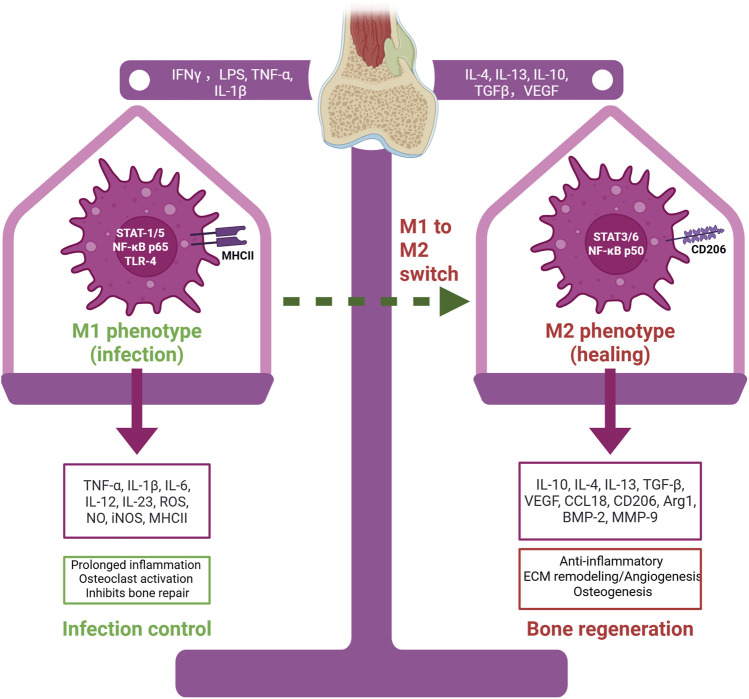
Macrophage polarization in infection-associated bone defects. M1 macrophages, activated by IFN-γ, LPS, TNF-α, and IL-1β, release pro-inflammatory mediators that control infection but inhibit bone repair. M2 macrophages, induced by IL-4, IL-13, IL-10, TGF-β, and VEGF, secrete anti-inflammatory and regenerative factors that promote tissue remodeling, angiogenesis, and osteogenesis. The M1–M2 switch is essential for effective bone healing. Created with BioRender.com.

There is an emerging view of specialized “osteoimmune” coupling, wherein macrophages directly influence bone-resorbing and bone-forming cells (Yao et al., 2021). For example, pro-inflammatory M1 macrophages can stimulate osteoclast differentiation by releasing TNF-α and other factors that upregulate RANKL on stromal cells, tipping the balance toward bone resorption (Hu et al., 2023). At the same time, M1 macrophage-derived cytokines inhibit osteoblastic activity and matrix mineralization, linking chronic inflammation to bone loss (Zhou and Hu, 2024). Beyond these direct effects on bone cells, macrophages also communicate with MSCs and endothelial cells. M2-polarized macrophages have been shown to reduce apoptosis of MSCs and stimulate their osteogenic differentiation via paracrine signaling–for instance, by activating the TGF-β/Smad pathway in MSCs (Shin et al., 2021; Xu et al., 2020). They also secrete matrix metalloproteinases and other remodeling enzymes that help form the extracellular matrix for new bone (Khoswanto, 2023). Intriguingly, timing appears to be crucial: if M2 polarization is induced too early (before effective clearance of debris or pathogens), healing may be impeded by curtailing necessary inflammation or prematurely suppressing angiogenesis. Conversely, if the inflammatory M1 phase persists for too long without switching, it can lead to fibrotic tissue formation or non-union of the bone. Thus, a harmonious temporal sequence of macrophage activation–an initial M1 surge followed by a timely transition to M2 – is considered essential for optimal bone regeneration (Figure 1) (Huang et al., 2022).

The importance of macrophages in bone repair is further underscored by the foreign body response to implants or scaffolds (Saleh and Bryant, 2018; Martin and García, 2021). When a biomaterial is implanted into bone (or any tissue), macrophages are among the first responders that determine the subsequent healing outcome (Niu et al., 2021). An unfavorable response dominated by M1 activity can lead to chronic inflammation, fibrous encapsulation of the implant, or osteolysis around the implant. In contrast, biomaterials that promote an M2-dominant response tend to integrate better and facilitate tissue regeneration. This concept has driven the development of osteoimmunomodulatory biomaterials–materials specifically designed to modulate the immune reaction for improved bone repair (Whitaker et al., 2021; Chen et al., 2023; Sanati et al., 2025). In addition, the degradation products of biomaterials (such as ions released from bioactive glasses or calcium phosphates) can have immunomodulatory effects. For example, ions like silicate or calcium released from certain bioactive materials have been found to attenuate inflammatory signaling and support tissue repair by acting on immune cells (Zheng et al., 2021; Zhong et al., 2022).

In summary, macrophages serve as a bridge between the immune system and the skeletal system during regeneration. Their polarization state is a master regulator of the inflammatory milieu, which in turn dictates whether bone healing will proceed smoothly or be derailed by chronic inflammation. A balanced macrophage response eliminates pathogens and damaged tissue (via M1 functions) and then shifts to support new bone formation (via M2 functions). This knowledge provides a compelling rationale for therapeutic strategies that target macrophage polarization. By steering macrophages toward a pro-healing phenotype at the appropriate time, it may be possible to enhance bone regeneration, especially in compromised scenarios. In the next sections, we will explore how this principle is being applied using engineered hydrogels to treat bone defects complicated by infection—a context in which macrophage behavior is even more critical and challenging to manage. These representative studies illustrate the key macrophage phenotypes, their stimuli, roles in bone healing, and impact in infection (Table 1).

**TABLE 1:** Macrophage phenotypes.

Phenotype	Key stimuli and cytokines	Roles in bone healing	Impact in infection	Refs
M1	IFN-γ, LPS; TNF-α, IL-1β, IL-6	Pathogen clearance; recruit cells	Chronic activation → inflammation; inhibits healing	Lu et al. (2017)
M2a	IL-4, IL-13; IL-10, TGF-β, VEGF	Angiogenesis; ECM deposition; osteogenesis	Blocked in chronic infection	Wang et al. (2017)
M2b	IC immune complexes; IL-1β, TNF-α	Mixed inflammatory/regenerative	May prolong inflammation	Gordon et al. (2014)
M2c	IL-10; IL-10, TGF-β	ECM remodeling	Delayed in osteomyelitis	Muñoz et al. (2020)
M1→M2	Seq. IFN-γ→IL-4	Optimal bone healing	Biofilm blocks transition	Huang et al. (2022)
Infect.-driven M1	Biofilms; TNF-α, IL-1β	Osteoclast activation	Common in MRSA	Zhu et al. (2019)
Infect.-suppressed M2	Bacterial toxins; ↓IL-10	Impaired regeneration	Delays healing	Sahin et al. (2017)
Hybrid M1/M2	Mixed cytokines	Balance inflammation/regeneration	Unstable phenotype	Porcheray et al. (2005)

## 3 Impact of infection on bone healing and immune dynamics

In the presence of a bone infection, the normal progression of healing described above is profoundly disturbed. Infection introduces a persistent source of pathogenic stimuli that keeps the immune system in a heightened state of inflammation. Instead of inflammation resolving after a few days, an active infection can sustain an M1-dominated environment for weeks or longer, thereby impairing the transition to the regenerative phase (Jin et al., 2018; Newman et al., 2021; Schlundt et al., 2021). Clinically, this manifests as delayed union or non-union of fractures, extensive bone loss (osteolysis), and the formation of sequestra (dead bone segments) in chronic osteomyelitis. The pathological hallmark of chronic osteomyelitis is a smoldering inflammatory response with infiltrates of neutrophils, macrophages, and lymphocytes surrounding infected bone tissue and any implanted materials (Jensen et al., 2024). Bacteria such as *S. aureus* can survive intracellularly within osteoblasts or hide in biofilms on bone surfaces, evading immune clearance (Wen et al., 2020). These bacteria continuously stimulate macrophages and other immune cells through pathogen-associated molecular patterns (PAMPs), causing ongoing production of pro-inflammatory cytokines and proteolytic enzymes that damage bone tissue (Shi and Xianlong, 2012). Additionally, bacterial toxins and virulence factors (for instance, *S. aureus* α-toxin, Panton–Valentine leukocidin (PVL), protein A, etc.) can directly induce apoptosis or dysfunction of osteoblasts and MSCs, further hindering bone formation.

One consequence of chronic infection is the disruption of the normal coupling between bone resorption and bone formation. Inflammatory cytokines like IL-1, IL-6, and TNF-α–abundantly produced in infected bone–strongly promote osteoclast differentiation and activity (Shaw and Gravallese, 2016). They do this by increasing RANKL expression on osteoblast-lineage and stromal cells and by priming osteoclast precursors. As a result, bone resorption is accelerated at infected sites, leading to bone loss (Zhou and Graves, 2022; Campbell et al., 2024). Meanwhile, factors crucial for bone formation (such as Wnt signals or BMP pathways) are suppressed by the inflammatory milieu. The net effect is an “uncoupled” remodeling: bone destruction proceeds unchecked while new bone formation is minimal. Macrophages in an infected environment often exhibit an M1 phenotype characterized by high IL-1β, TNF-α, and inducible nitric oxide synthase (iNOS) expression, because they are continuously triggered by bacterial components (Zhu et al., 2019). Normally, as inflammation subsides, macrophages would switch to an M2 phenotype and release factors like IL-10 and VEGF to promote repair. However, in the face of persistent infection, this switch is inhibited or significantly delayed (Sahin et al., 2017). Indeed, persistent pro-inflammatory macrophage polarization impairs both angiogenesis and osteogenesis, two processes essential for effective bone regeneration (Zhang Z. et al., 2023). For example, in a mouse model of a contaminated fracture, an abundance of M1 macrophages correlated with poor vascularization of the callus and failure of bony bridging. By contrast, treatments that reduced inflammation and supported M2 polarization led to improved blood vessel formation and bone repair (Hozain and Cottrell, 2020).

Neutrophils and T-cells also interact with macrophages in the infected bone milieu to shape outcomes. Neutrophils are first responders to infection and release proteases and ROS to kill bacteria, but excessive neutrophil activity can damage bone tissue and generate signals that perpetuate macrophage activation (Nguyen et al., 2017). Neutrophils also form neutrophil extracellular traps (NETs) in chronic infection, which have been linked to inflammatory bone loss in diseases like osteomyelitis and peri-prosthetic joint infection (Castanheira and Kubes, 2019; Carmona-Rivera et al., 2024). T-lymphocytes further modulate macrophage behavior: Th1 cells secrete IFN-γ that reinforces M1 polarization, whereas Th2 cells produce IL-4 and IL-13 that promote M2 polarization (Shapouri-Moghaddam et al., 2018). In chronic infections, a bias toward Th1 responses is often observed, supporting the M1-dominated state. Additionally, regulatory T cells (Tregs) may become dysregulated; normally Tregs help suppress excessive inflammation and support repair, but in some chronic infection scenarios an “exhausted” or ineffective Treg response contributes to uncontrolled inflammation (Richert-Spuhler and Lund, 2015).

Another layer of complexity is introduced by bacterial biofilms on implants or bone surfaces. Biofilm-encased bacteria are shielded from both antibiotics and immune cells. Macrophages attempting to phagocytose biofilm bacteria often fail, leading to “frustrated phagocytosis” – they remain in an activated state, releasing inflammatory mediators but unable to clear the infection (Arciola et al., 2023; Li M. et al., 2023). Biofilm fragments can periodically disperse, causing recurrent acute inflammatory flares. The immune system’s inability to resolve a biofilm infection means the local environment is locked in a state of chronic inflammation, significantly impairing healing (Mendhe et al., 2023; Mitrova Telenta et al., 2025). Clearly, an active infection skews the immune response into a pathological loop that must be interrupted to allow bone regeneration to proceed.

Given these insights, it has become evident that treating an infection-associated bone defect is not only about killing the bacteria but also about recalibrating the immune environment. Traditional antibiotic therapy, while essential, may not fully address the inflammatory imbalance or the tissue damage that has occurred. Thus, there is a strong rationale for adjunctive therapies that modulate the immune response. One approach is local delivery of anti-inflammatory or immunomodulatory agents to temper the M1 response and encourage an M2 shift once the bacterial load is reduced. For example, anti-TNF or anti-IL-1 therapies (commonly used in rheumatoid arthritis) have been proposed to reduce infection-induced bone loss (Dong et al., 2021; Su et al., 2022). Another approach is to strategically time the delivery of immunomodulators: initially allowing or even promoting some pro-inflammatory activity to fight infection, then switching to pro-healing signals. In essence, the goal is to mimic the natural healing sequence that the infection has stalled.

This is where biomaterials–especially hydrogels–can play a transformative role. Hydrogels can serve as vehicles for combination therapy, simultaneously delivering antibiotics to eliminate bacteria and immune-regulating molecules to modulate inflammation (Kaur et al., 2024; Ahmed et al., 2025). Furthermore, an ideal biomaterial for infected bone regeneration would itself be “immune-instructive,” meaning that its intrinsic properties help quell harmful inflammation and support constructive immune reactions (Negrescu and Cimpean, 2021).

## 4 Design of immunomodulatory hydrogels for bone regeneration

Hydrogels are three-dimensional polymer networks capable of holding a large amount of water, and they can be formulated from various natural or synthetic polymers (e.g., collagen, hyaluronic acid, gelatin, polyethylene glycol, alginate, chitosan). In bone tissue engineering, hydrogels offer a conducive microenvironment for cell encapsulation and growth factor delivery, and they can be engineered to degrade at rates matching new tissue formation (Cota Quintero et al., 2025). The emerging paradigm of immunomodulatory hydrogels goes a step further by incorporating cues that actively steer the host immune response. The design of such hydrogels involves multiple considerations: the choice of base material, mechanical properties (stiffness, viscoelasticity), biodegradability, and the inclusion of bioactive factors (cytokines, drugs, peptides, or nanoparticles) that influence immune cells, particularly macrophages (Zhang Z. et al., 2023; Nie et al., 2024; Lai et al., 2025).

### 4.1 Base materials and intrinsic immunomodulatory properties

Natural polymer hydrogels (for example, those derived from decellularized extracellular matrix (ECM), fibrin, or hyaluronic acid) often inherently support a more regenerative immune response compared to purely synthetic materials (Mehrban et al., 2020). For instance, hydrogels made from decellularized bone matrix or collagen can provide biochemical signals that favor a healing phenotype–these materials tend to elicit a type 2 (M2-like) immune response characterized by upregulation of IL-4 and IL-10 (Mehrban et al., 2020). In contrast, certain unmodified synthetic hydrogels (e.g., high-stiffness polyacrylamide gels) might provoke a foreign body reaction skewed toward M1 macrophages and fibrosis (Zhuang et al., 2020). Therefore, many strategies combine natural and synthetic components to achieve both favorable bioactivity and tunable properties (Sergi et al., 2020). Hyaluronic acid (HA) itself can engage cell surface receptors (CD44) on immune cells: high-molecular-weight HA tends to be anti-inflammatory, whereas fragmented HA can be pro-inflammatory. Thus, HA-based hydrogels are often designed using a high molecular weight form to avoid triggering M1 macrophage activation (Oates et al., 2024). Chitosan, a polysaccharide derived from chitin, has intrinsic antimicrobial properties and can activate immune cells; interestingly, appropriately formulated chitosan hydrogels have been shown to reduce inflammatory cell infiltration and support M2 polarization, partly by scavenging toxic byproducts like ROS and through cationic interactions with cell membranes (Ghattas et al., 2025). Gelatin (denatured collagen) hydrogels present RGD motifs that promote cell adhesion and can be crosslinked under mild conditions to encapsulate cells or therapeutic factors. Gelatin-based hydrogels are generally biocompatible and have been observed to cause only transient inflammation that transitions to healing—especially when loaded with bioactive molecules (Mohanto et al., 2023; Zhai et al., 2023).

### 4.2 Mechanical and structural cues

The physical properties of hydrogels significantly influence macrophage responses. Macrophages are mechanosensitive; they sense the stiffness of their substrate through integrin-mediated adhesion and adjust their phenotype accordingly. Studies have indicated that softer matrices (with an elastic modulus in the range of healthy soft tissue) often encourage macrophages to adopt an M2-like gene expression profile, whereas very stiff matrices (comparable to glass or rigid plastic) favor an M1 profile (Li Y. et al., 2023; Nie et al., 2024). In bone tissue engineering, hydrogels must often be reinforced or combined with inorganic components (like hydroxyapatite particles or bioactive glass) to provide adequate mechanical support and osteoconductivity. Impressively, these mineral additions can also modulate immune responses–for example, incorporating nano-hydroxyapatite or calcium silicate into hydrogels has been shown to decrease pro-inflammatory cytokine secretion by macrophages and upregulate markers associated with healing. In general, designing hydrogels with an interconnected porosity that permits cell infiltration and waste diffusion is beneficial for avoiding chronic inflammation and hypoxic conditions that can exacerbate M1 activation.

### 4.3 Incorporation of immunomodulatory factors

One of the most direct ways to create an immunomodulatory hydrogel is to load it with cytokines, chemokines, or drugs that influence macrophage polarization (Lai et al., 2025). Immunomodulatory hydrogels have emerged as a promising approach for chronic wound healing and tissue regeneration by controlling macrophage polarization. Such hydrogels can be designed to incorporate specific cytokines, chemokines, or pharmaceutical agents that push macrophages toward a desired phenotype. The physical and chemical properties of hydrogels—composition, stiffness, surface morphology—can also be tailored to regulate macrophage behavior and promote tissue repair (Zhuang et al., 2020). Studies have demonstrated the efficacy of cytokine-loaded hydrogels in modulating macrophage polarization and enhancing healing in various tissues, including skin, bone, and cartilage (Barthes et al., 2021; Giri and Rath, 2024). Furthermore, encapsulating monocytes within hydrogels that contain anti-inflammatory cytokine cocktails can create a personalized implant interface, potentially improving biocompatibility and reducing adverse immune responses (Barthes et al., 2021).

### 4.4 Immune cell delivery

Another dimension of immunomodulatory design is the delivery of actual immune cells with the hydrogel. Exploratory approaches have loaded hydrogels with exogenous macrophages or monocytes that have been pre-programmed to a desired phenotype (for example, *ex vivo* polarized M2 macrophages) (Zhang Z. et al., 2023). These cells can potentially augment the host response by secreting beneficial cytokines or by replacing dysfunctional host macrophages in chronic infection scenarios (Tu et al., 2021). Alternatively, MSCs—which are known for their immunomodulatory capacity—are often delivered via hydrogels; MSCs can secrete anti-inflammatory factors and extracellular vesicles that induce M2 polarization of host macrophages (Arabpour et al., 2021). The combination of MSCs and immunomodulatory hydrogels can be synergistic: the hydrogel creates a niche that supports the MSCs and tunes the immune response, while the MSCs secrete factors that further calm inflammation and promote repair (Cheng et al., 2024).

### 4.5 Biomaterial surface ligands and patterning

Hydrogels can be functionalized with specific ligands that interact with macrophage receptors to influence their behavior (Xu et al., 2021). One such target is the macrophage mannose receptor (CD206), which is highly expressed on M2 macrophages. By presenting mannose or mannose-mimetic peptides on a hydrogel, some studies have aimed to preferentially recruit, engage, and stabilize M2 macrophages at the material surface (Kaps et al., 2020). Conversely, masking or reducing the exposure of “danger” signals (for example, certain exposed polymer chemical groups that trigger Toll-like receptors) can prevent excessive M1 activation. As an example, one study showed that hydrogels with an optimal density of the adhesive peptide RGD (arginine-glycine-aspartate) promoted better macrophage adhesion and a balanced cytokine profile, whereas hydrogels lacking cell-adhesive cues kept macrophages in suspension and resulted in higher M1 cytokine release (Cha et al., 2017). The spatial distribution of cues can also be important: some hydrogels have core–shell structures or built-in gradients where, for instance, the outer layer presents an antibacterial, M1-promoting agent (to manage infection at the interface), and the inner core presents an M2-promoting factor for the healing phase (Xiao et al., 2023).

**FIGURE 2:**
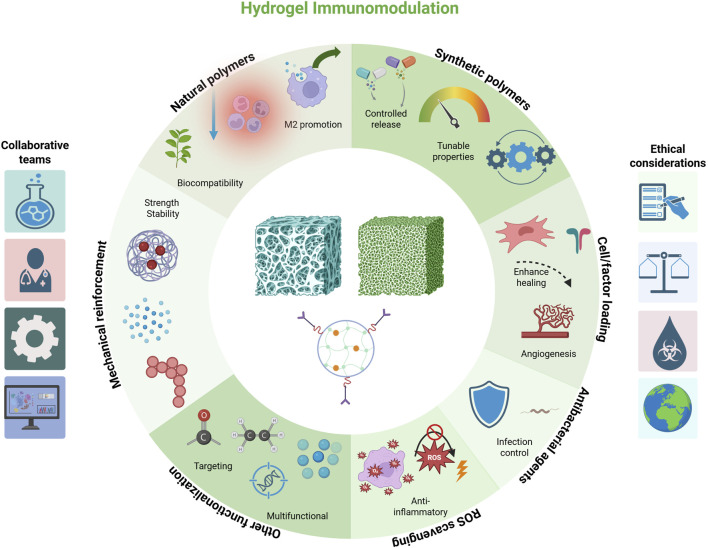
Schematic representation of key design approaches for immunomodulatory hydrogels, including natural polymers, synthetic polymers, mechanical reinforcement, cell/factor loading, ROS scavenging, antibacterial agents, and other functionalization. Each strategy is associated with representative functions such as biocompatibility, controlled release, immune modulation, infection control, and targeted or multifunctional effects. Created with BioRender.com.

In summary, the design toolbox for immunomodulatory hydrogels is rich and multifaceted. By carefully selecting material composition, mechanical properties, and bioactive payloads, researchers have created hydrogel systems that not only serve as scaffolds for new bone growth but also actively guide the immune processes underlying successful regeneration. These hydrogels aim to recreate the conditions of normal bone healing even in adverse settings–quieting excessive inflammation and encouraging the body’s natural repair machinery. In the context of infection-associated defects, however, an additional key requirement is antimicrobial efficacy. We thus turn our attention to how hydrogels can be equipped to handle bacterial challenges while simultaneously performing immunomodulation. These representative studies illustrate diverse hydrogel designs aimed at modulating immune responses in infected bone defects (Table 2; Figure 2).

**TABLE 2:** Hydrogel design strategies.

Strategy	Example	Immunomodulation	Pros and cons	Refs
Natural polymers	Collagen, HA, gelatin	M2 promotion	Biocompatible; variable	Mehrban et al. (2020)
Synthetic polymers	PEG, PCL, PLGA	Tunable mechanics	Reproducible; inert	Sergi et al. (2020)
Mech. reinforcement	Nano-HA, bioactive glass	Ion-mediated M2	Osteoconductive; stiff	Li et al. (2023b)
Cytokine loading	IL-4, IL-10, TGF-β	Phenotype switch	Potent; short life	Zou et al. (2021)
Cell delivery	MSCs, M2 macrophages	Paracrine M2	Strong; complex	Cheng et al. (2024)
ROS scavenging	Ceria, tannic acid	Reduce stress	Anti-inflam; no killing	Qi et al. (2022)
Ligand target	Mannose-CD206	Recruit M2	Specific; density control	Kaps et al. (2020)
Sequential	IFN-γ→IL-4 + antibiotics	Timed switch	Mimics heal; complex	Chen et al. (2018)

## 5 Hydrogels for concurrent antibacterial and immunomodulatory therapy

**FIGURE 3:**
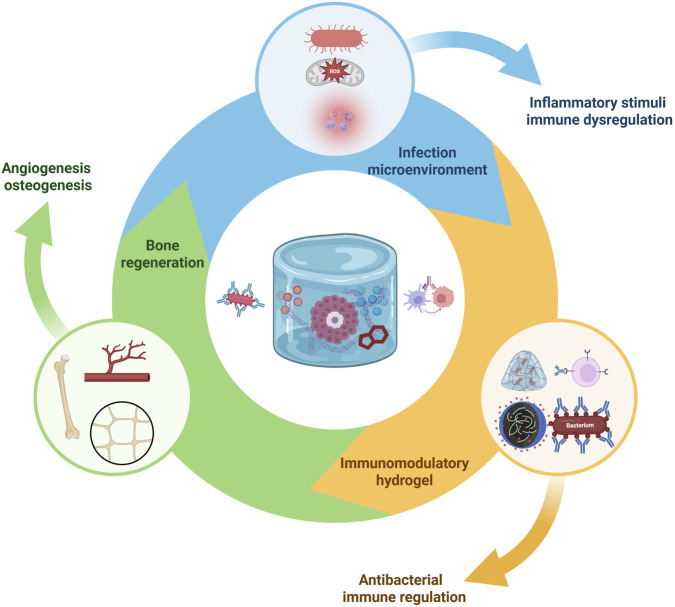
Immunomodulatory hydrogel-mediated antibacterial and bone regenerative mechanisms. Infection microenvironments characterized by bacteria, ROS, and inflammatory cells trigger immune dysregulation. Multifunctional hydrogels deliver antibacterial agents and immunomodulatory cues to suppress pathogens, modulate immune responses, and promote osteogenesis and angiogenesis, resulting in enhanced bone regeneration in infection-compromised conditions. Created with BioRender.com.

Treating infected bone defects demands a dual functionality: eliminating or controlling the infection and promoting tissue regeneration. Hydrogels provide a platform where both antibacterial and immunomodulatory strategies can be integrated. This section discusses various approaches to confer antibacterial activity to hydrogels, and how these approaches interface with the immune-modulating aspects. The interplay between infection microenvironments, immune dysregulation, and bone regeneration can be effectively addressed by multifunctional immunomodulatory hydrogels (Figure 3).

In addition to exogenous payloads, several hydrogel backbones display intrinsic anti-inflammatory activity. A representative example is high–molecular-weight hyaluronic acid (HA), whose engagement of receptors such as CD44 can dampen pro-inflammatory signaling, reduce neutrophil recruitment, and favor pro-regenerative macrophage states. Such “drug-free” immunomodulation can be leveraged in defect-filling intralesional systems when antibiotic load must be minimized or combined with low-dose antimicrobials. Effects are context- and molecular-weight–dependent—low-molecular-weight fragments may behave differently—so material specification and degradation profile should be reported (Li Y. et al., 2023).

Across the cited studies, osteomyelitis hydrogels were evaluated in standard contaminated-defect and implant-associated models (rat/mouse segmental defects, intramedullary-canal infections with colonized wires/pins, and calvarial critical-size defects), using local delivery (intralesional, intramedullary, or subperiosteal/topical) and common endpoints (quantitative cultures, μCT bone metrics, and histology).

### 5.1 Antibiotic-loaded hydrogels

One straightforward approach is loading conventional antibiotics into hydrogels for local release. Local antibiotic delivery achieves high drug concentrations at the infection site without systemic toxicity, and several clinical products already exist (for example, antibiotic-impregnated bone cement or calcium sulfate beads) (Alt et al., 2015). Hydrogels can be advantageous in this context because they are often biodegradable and can fill irregular defects. Antibiotics like vancomycin, gentamicin, tobramycin, and ciprofloxacin have been successfully incorporated into various hydrogel formulations (Shukla and Shukla, 2018; Sun et al., 2024b). For instance, a thermosensitive chitosan-based hydrogel carrying vancomycin nanoparticles was developed to treat bone infections: it remained liquid at room temperature for injectability and gelled at body temperature, releasing vancomycin in a sustained manner to eradicate *S. aureus*, while the chitosan matrix itself helped modulate the local immune response (Hoque et al., 2017). Importantly, the immunomodulatory hydrogel paradigm emphasizes that simply delivering antibiotics may quell infection, but if the inflammatory environment is not also addressed, healing may still stall (Qi et al., 2024). Therefore, researchers have combined antibiotics with immune cues. For example, several studies have developed hydrogels loaded with interleukin-4 (IL-4) to promote M2 macrophage polarization, which in turn reduces inflammation and accelerates tissue repair. These IL-4-loaded hydrogels have shown promising results in enhancing osteogenesis and bone regeneration (Zou et al., 2021; Zhao et al., 2024; Chen Y. et al., 2025). The timing and release profile are critical: ideally, the antibiotic is released aggressively early on to knock down bacterial counts, whereas the immunomodulator is co-released or slightly delayed to avoid suppressing the initial needed inflammation. Advanced release systems often employ stimuli-responsive mechanisms to achieve this staged delivery (Sikder et al., 2021; Nazir et al., 2022). After the infection is under control, the remaining hydrogel can degrade to release factors that aid healing. The approaches listed provide an overview of antibacterial components integrated into hydrogels and their associated immune-modulating effects (Table 3). Model note: typically rat/mouse segmental-defect osteomyelitis with *S. aureus*; intralesional delivery after debridement; endpoints include CFU, μCT, and histology.

**TABLE 3:** Antibacterial components and modes.

Antibacterial	Example	Immune link	Pros and cons	Refs
Antibiotics	Vancomycin, gentamicin	Limited	Familiar; resistance	Hoque et al. (2017)
Metal NPs	Ag, Cu, ZnO	M2 skew (dose-dependent)	Broad; toxic risk	Jiang et al. (2020)
AMPs	LL37, engineered peptides	Cytokine modulation	Low resistance; unstable	Chen et al. (2025b)
Photothermal	PDA, Au nanorods	↓M1; biofilm killing	On-demand; device	Wu et al. (2023)
Photodynamic	Porphyrins, RB	ICD	Precise; depth limit	Klausen et al. (2020)
ROS + kill	Ceria + antibiotics	M2 promotion	Dual; slow onset	Qi et al. (2022)
Bioactive ions	Ca, Si, Zn	M2 + bone regen	Multi; dose ctrl	Huo et al. (2024)

### 5.2 Antimicrobial nanoparticles and ions

Beyond traditional antibiotics, hydrogels have been infused with antimicrobial nanoparticles such as silver (Ag), copper (Cu), zinc oxide (ZnO), or nanoscale photocatalysts (Tomić and Vuković, 2022). Silver nanoparticles (AgNPs) are well-known for broad-spectrum antimicrobial action via multiple mechanisms (e.g., disrupting bacterial membranes, generating ROS, and damaging bacterial DNA) and have been widely incorporated into biomaterials. Hydrogels containing AgNPs can continuously release silver ions that kill bacteria; interestingly, low levels of silver ions have also been reported to attenuate inflammatory cytokine production by macrophages (perhaps by inhibiting certain signaling pathways), which might help reduce excessive inflammation (Jiang et al., 2020; Albao et al., 2024; Rodrigues et al., 2024). Copper ions or nanoparticles are another addition: Cu has antimicrobial properties and also pro-angiogenic effects (copper can induce new blood vessel formation by stabilizing HIF-1α and upregulating VEGF) (Rigiracciolo et al., 2015). For example, a conductive hydrogel containing copper nanoparticles not only eradicated bacteria in an infected bone model but also influenced macrophage polarization toward an M2 phenotype, likely through copper’s involvement in cellular signaling and oxidative stress modulation (Zha et al., 2023; Yang et al., 2025). Incorporation of bioactive glass or calcium-based nanoparticles endows hydrogels with the release of ions like Ca^2+^, silicate (SiO_4_^4-^), or Zn^2+^, which can inhibit bacterial growth and at the same time modulate bone cells and immune cell functions (Keenan et al., 2016; Huo et al., 2024). For example, zinc has mild immunomodulatory effects–zinc oxide nanoparticles in a hydrogel can kill bacteria and have been observed to reduce the expression of pro-inflammatory mediators by macrophages (Huo et al., 2024). Model note: often implant-associated infection (biofilm-coated K-wire/pin) or surface-contaminated defects; subperiosteal/topical or intralesional delivery; endpoints include bacterial log-reduction, dosimetry/safety, and histology.

### 5.3 Photothermal and photodynamic hydrogels

New strategies harness external stimuli like light to trigger antimicrobial activity. Hydrogels containing photothermal agents (e.g., gold nanorods, polydopamine, or black phosphorus nanosheets) can generate heat upon near-infrared (NIR) light irradiation, effectively killing bacteria in the vicinity (Li et al., 2024b; Sun et al., 2024a). Such photothermal hydrogels have been tested in infected bone defect models–a mild local heating can ablate bacteria (including those within biofilms) and, interestingly, moderate heat (around 45 °C–50 °C for short durations) can also induce a kind of “heat shock” response in immune cells that skews them away from a strongly pro-inflammatory state (since extreme inflammatory enzymes are heat-labile) (Zhang et al., 2024; Wu et al., 2025). For instance, one study created a hydrogel with polydopamine-coated bioglass; upon NIR exposure, it produced heat and also released bioactive ions faster–this combination eradicated *S. aureus* and led to an M2-rich environment with high levels of TGF-β and IL-10, promoting bone regeneration (Wu et al., 2023). Photodynamic therapy (PDT) is another approach, where hydrogels incorporate photosensitizers (e.g., porphyrins) that generate cytotoxic singlet oxygen and free radicals under light to kill microbes. In one example, a hydrogel with a photosensitizer and an immune-tolerant polymer backbone was used to disinfect bone infection sites via light activation; concurrently, the polymer degraded to release an anti-inflammatory agent (Klausen et al., 2020). Although light-based methods require external equipment and careful control to avoid tissue damage, they offer temporal control: one could perform a photothermal or PDT treatment early to sterilize the area, and afterwards the hydrogel remains in place as a scaffold releasing growth factors or cytokines for healing. Model note: evaluated in implant-associated and contaminated-defect models; delivery is intralesional or subperiosteal/topical; endpoints include bacterial log-reduction, tissue safety, and histology.

Broadly, three strategy families have emerged. Antibiotic-releasing hydrogels deliver high local drug levels with clinical familiarity and straightforward QA, but face elution-lifetime limits and resistance pressure. Photothermal/photodynamic antimicrobial hydrogels can eradicate biofilm-associated and drug-tolerant bacteria with spatiotemporal control, yet require light access and careful dosimetry to avoid collateral thermal/photosensitizer injury. Nano-enabled immunomodulatory hydrogels couple pathogen killing with macrophage reprogramming and microenvironment repair, offering durability and host-directed benefits, while demanding rigorous safety profiling and scalable manufacturing. These trade-offs explain why defect-filling intralesional systems are most compelling for post-traumatic osteomyelitis, whereas topical/subperiosteal layers may better suit ischemic wounds; representative recent reports span nanocomposite hydrogels for infected wounds, macrophage-targeted platforms, and macrophage-mediated immunomodulation in bone repair (Mu et al., 2025; Guo et al., 2024; Bi et al., 2024).

### 5.4 Reactive oxygen species (ROS) scavenging and immune modulation

In infected and inflamed tissues, excessive ROS (from neutrophils and M1 macrophages) cause tissue damage and prolong inflammation. Some hydrogels are specifically designed to scavenge ROS as a way to both protect host cells and signal macrophages to calm down. Antioxidant components such as cerium oxide nanoparticles, bioactive polyphenols (e.g., curcumin or tannic acid), or enzyme mimetics (like catalase- or superoxide dismutase-mimicking compounds) have been integrated into hydrogels (Qi et al., 2022). By reducing ROS, these hydrogels mitigate oxidative stress on bone cells and also indirectly push macrophages towards an M2 state (since high ROS levels favor M1 activation) (Chen et al., 2022; Qi et al., 2022). Recent studies have explored ROS-scavenging hydrogels for bone regeneration and wound healing. These hydrogels can reduce inflammation, enhance osteogenesis, and promote tissue repair by eliminating excess ROS at injury sites (Huang et al., 2025; Sun et al., 2023). Various approaches have been developed, including incorporating ROS-responsive nanoparticles, modifying polymer chains to have antioxidant properties, and using ROS-labile linkers in the hydrogel network (Sun et al., 2023). Such hydrogels have shown efficacy in treating bone defects, diabetic wounds, and periodontitis by reducing pro-inflammatory cytokines, guiding macrophage polarization, and enhancing osteoblast activity. Some designs also allow for controlled release of therapeutic agents in response to local ROS levels (Peng et al., 2024). Overall, ROS-scavenging hydrogels demonstrate significant potential for improving outcomes in inflammatory and degenerative conditions by modulating the local microenvironment. While ROS scavenging itself is not directly antibacterial, it can be paired with a mild antimicrobial component and primarily serves to protect host tissue and resolve inflammation once the active infection is controlled (Li et al., 2024a).

### 5.5 Sequential or stage-wise therapies

One particularly elegant strategy with hydrogels is to achieve a staged therapeutic response that aligns with the needs of each phase of healing (Chen J. et al., 2025). For instance, an injectable hydrogel microsphere system was devised in which microspheres loaded with calcitriol (vitamin D_3_) create an initial anti-inflammatory milieu that pre-conditions the site; then the bulk hydrogel releases BMP-2 at a later time to stimulate bone formation (Chen J. et al., 2025). In an infected context, sequential delivery might involve an initial release of an antimicrobial agent (or even an M1-stimulating signal to assist early infection clearance) followed by a delayed release of immunoresolving agents. Chen et al. (2018) described an *in vivo* study with a hydrogel that sequentially delivered first IFN-γ (for 2 days) and then IL-4 (from days 4–14) in a contaminated bone defect. The result was effective bacteria clearance with a strong early inflammatory response that was then purposefully switched to an anti-inflammatory mode, leading to significantly improved bone regeneration compared to either constant release of both factors or no immunomodulator at all. This underscores that timing in immunomodulation is as important as the agents themselves.

The combined use of antibacterial and immunomodulatory features in hydrogels has shown great promise in preclinical studies (Han et al., 2024; Qi et al., 2024). A recent example by Du et al. (2024) encapsulates this concept: they created a multifunctional injectable hydrogel (based on oxidized HA crosslinked with the phenolic compound rosmarinic acid and reinforced with laponite nanoclay). This hydrogel provided a self-reinforced matrix with good mechanical properties for a load-bearing defect, exhibited potent antibacterial effects against both *S. aureus* and *E. coli* (thanks to the released rosmarinic acid and the inherent clay, which can bind bacterial membrane components), and critically, it induced M2 polarization of macrophages while also promoting osteogenic differentiation of MSCs. In infected rat skull defects, the hydrogel significantly accelerated bone healing without systemic toxicity, highlighting how multi-functional components (antioxidant, antibacterial, immunomodulatory) can act in concert. Another recent study by Yu et al. (2025) extended this strategy by developing an injectable dual-network hydrogel specifically for treating osteomyelitis. This advanced hydrogel acts as an effective adsorbent of bacteria and inflammatory factors, employing a dual-crosslinking mechanism to reinforce the hydrogel structure after injection. By efficiently adsorbing bacteria and inflammatory cytokines from the infected microenvironment, the hydrogel creates an ideal antibacterial and anti-inflammatory interface at the bone–hydrogel junction, greatly facilitating bone regeneration and healing. These representative studies highlight how hydrogels can be engineered to integrate antibacterial and immunomodulatory functions for infection-associated bone regeneration (Table 4).

**TABLE 4:** Representative preclinical studies.

Hydrogel	Antibacterial	Immune strategy	Model	Outcome	Refs
Chitosan + VAN NPs	Vancomycin	ROS scav	Rat tibia	Clear MRSA; ↑M2	Hoque et al. (2017)
CuNP hydrogel	CuNPs	M2 + angiogenesis	Rat skull	↓Inflam; ↑Vessels	Zha et al. (2023)
AgNP hydrogel	AgNPs	Cytokine mod	Rabbit defect	Antibacterial; integrate	Jiang et al. (2020)
HA + rosmarinic + laponite	Phenolic acid	Antioxidant + M2	Rat skull	Kill + osteogenic	Du et al. (2024)
Seq. IFN-γ/IL-4 hydrogel	—	M1→M2 timed	Rat tibia	Control inf.; repair	Chen et al. (2018)
Bioactive glass GelMA	Ion release	M2 + osteogenesis	Mouse skull	Integrate; ↓fibrosis	Li et al. (2023b)
ROS-scav. polyphenol hydrogel	Tannic acid	ROS neutral	Rat femur	↓ROS; ↑angiogenesis	Qi et al. (2022)

Some studies (e.g., Huo et al., 2024; Qi et al., 2022) used infectious skin wound models rather than bone defect models; their inclusion here serves as mechanistic evidence for infection-associated bone regeneration.

In conclusion, hydrogels can be engineered as all-in-one solutions that address both major hurdles in infected bone repair: infection control and immune modulation. By tailoring release profiles and material characteristics, these systems aim to first act as an antimicrobial depot to sterilize the wound and then seamlessly transition into a regenerative scaffold that encourages the body’s healing processes. While complex in design, such hydrogels have shown in preclinical models that they can reduce bacterial load, modulate macrophages from an M1-to M2-dominated environment, enhance angiogenesis, and ultimately result in more robust and faster bone regeneration compared to traditional approaches. These promising outcomes pave the way for translation, but there are still practical and regulatory challenges to overcome—which will be discussed in the following section.

## 6 From bench to bedside: challenges and prospects for clinical translation

Immunomodulatory, infection-fighting hydrogels for bone regeneration represent a cutting-edge convergence of bioengineering and immunotherapy. Translating these complex interventions from the laboratory to routine clinical use, however, is a non-trivial endeavor. Several key considerations must be addressed, including safety and biocompatibility, manufacturing consistency, regulatory approval pathways, and demonstrating efficacy in human patients.

### 6.1 Biocompatibility and safety

First and foremost, any hydrogel introduced into the body must be safe. While many of the base polymers used (collagen, gelatin, hyaluronic acid, polyethylene glycol) have good biocompatibility records, the addition of new bioactive agents (cytokines, nanoparticles, etc.) requires careful toxicity evaluation. For example, silver nanoparticles are effective antimicrobials but in high concentrations can be cytotoxic to host cells and have raised concerns about argyria or local tissue discoloration in other applications (Qing et al., 2018). Therefore, an optimal dosing must be found that is bactericidal yet not harmful to human cells. Similarly, cytokines like IFN-γ or IL-4 used in delivery systems need to be at appropriate doses: too much IFN-γ could trigger systemic inflammation or off-target effects, whereas too much IL-4 might suppress necessary early immune functions. Extensive biocompatibility testing in animal models is required to ensure that the hydrogel itself does not provoke an unintended chronic foreign body reaction or autoimmunity. An advantageous aspect of many immunomodulatory hydrogels is that they are actually designed to reduce inflammation–indeed, several have shown less fibrous capsule formation and better tissue integration than inert controls in animal studies. Nonetheless, subtle species differences exist; a material that modulates macrophages effectively in mice might behave differently in humans, where the immune system’s complexity is higher. Long-term degradation profiles of the hydrogel also need assessment. If a hydrogel degrades into byproducts (e.g., acidic oligomers in some polyesters), those byproducts should not accumulate or cause local pH drops that could ironically incite inflammation. Strategies like incorporating buffering molecules or using naturally metabolizable linkages (such as enzymatically cleavable peptides) can help ensure biocompatible degradation.

### 6.2 Manufacturing and reproducibility

Many immunomodulatory hydrogels involve sophisticated formulations with multiple components (for instance, a polymer backbone, a crosslinker, one or more types of nanoparticles, and two different cytokines). Scaling up the production of such complex materials while maintaining batch-to-batch consistency can be challenging. Pharmaceutical Good Manufacturing Practice (GMP) conditions require well-defined processes and rigorous quality controls. Natural materials can introduce variability (different batches of collagen, for example, might have varying gelation properties), which may necessitate using recombinant or synthetic analogs for consistency. Moreover, inclusion of biologics like growth factors or cytokines introduces storage and stability considerations–these proteins can denature or lose activity during sterilization (autoclaving is typically not possible for them, so alternative sterilization methods like filtration or gamma irradiation must be validated).

#### 6.2.1 Regulatory pathway

Combination products (part device, part drug/biologic) face a complex regulatory pathway. An immunomodulatory hydrogel that contains a known antibiotic might be viewed primarily as a drug-delivery device by regulators, whereas one that contains a new cytokine or a cell therapy component could be regulated as a biologic. These classifications affect the type of preclinical and clinical data required. Navigating whether the primary mode of action is considered pharmacological (drug) or mechanical/structural (device) is part of the regulatory strategy and will dictate whether approval goes through agencies’ device or biologic frameworks (or both).

### 6.3 Clinical trial Design and efficacy endpoints

Designing human trials for bone regeneration in the context of infection can be tricky. The patient population can be heterogeneous (for example, diabetic patients with foot osteomyelitis vs. patients with traumatic bone injuries vs. patients with revision arthroplasty infections). A clear use-case needs to be defined: is the hydrogel meant to replace part of standard care (e.g., used instead of systemic antibiotics), or is it an adjunct to standard care that might improve outcomes (e.g., applied after surgical debridement to reduce infection recurrence and boost healing)? Likely, initial trials would use these hydrogels as adjunctive therapy to ensure patients still receive standard-of-care antibiotics. Endpoints in bone regeneration trials include not just infection clearance (which can be measured by microbiological cultures, inflammatory markers, and absence of relapse over ∼1 year) but also bone healing metrics (such as radiographic evidence of defect fill, time to weight-bearing, and functional recovery scores). These trials may need to follow patients for many months (or even a year or more) given the slow nature of bone regeneration and the potential for late recurrence of infection.

### 6.4 Challenges of immune variability

Human immune responses are diverse. Factors like age, diabetes, smoking status, or genetic differences can affect how a patient’s macrophages respond to a given stimulus or biomaterial. This variability means that a hydrogel that works well in one patient group might have diminished effect in another, or could even elicit an unforeseen immune reaction in certain individuals. Tailoring treatments to patient-specific immune profiles (or designing broadly tolerable immunomodulatory cues) remains a challenge.

### 6.5 Cost and practicality

The cost of advanced biomaterials can be significant. Off-the-shelf simple materials (like antibiotic-infused bone cement) are relatively cheap. A hydrogel containing multiple recombinant factors or nanoparticles might be much more expensive. Health economic analyses will have to weigh the upfront cost of such a product against potential savings from reduced repeat surgeries, shorter hospitalization times, and improved limb salvage rates. Practically, surgeons will also need the product to be user-friendly–ideally injectable or moldable *in situ*, with a reasonable working time, and not too technically demanding to prepare during surgery. Many current research hydrogels are indeed formulated as injectable systems that gel within minutes at body temperature or upon mixing components, which aligns with surgical workflow requirements.

### 6.6 Regulatory snapshot

Most antimicrobial/immunomodulatory hydrogels will be regulated as combination products, with the primary mode of action (PMOA) determining the lead pathway. If the scaffold’s structural/space-filling function predominates, a device-led pathway applies (e.g., bench performance, sterilization validation, shelf-life, usability; biocompatibility and extractables/leachables per recognized standards; lot-to-lot controls for polymer chemistry/crosslinking). If the therapeutic effect is primarily driven by the active agent (antibiotic, biologic, or nanoparticle drug), a drug/biologic-led pathway applies (CMC for the active and the matrix, GLP tox/pharm, stability, and release specification). Across both routes, sterility assurance, release kinetics tied to clinically relevant acceptance criteria, and manufacturability/scale-up are pivotal. Early-phase trials should prioritize infection control (signs/cultures/reoperation), safety, and functional/union outcomes, using materials manufactured under conditions representative of the final product.

### 6.7 Context relative to non-hydrogel immunomodulation

Compared with systemic immunomodulators (e.g., steroids, biologics, small-molecule anti-inflammatories), hydrogel depots provide spatially confined, high local exposure within debrided defects, minimize off-target immunosuppression, and simultaneously manage dead space (Liu and Segura, 2020; Whitaker et al., 2021). Limitations include the need for placement (often surgical), dependence on debridement quality, and finite release lifetimes. Nanoparticle-only approaches (systemic or local) enable cell/organelle targeting and stimulus-responsive signaling, but without a scaffold they are prone to washout and short residence, offer no mechanical dead-space management, and may raise concerns about off-target accumulation and scale-up (Guo et al., 2024). Hydrogels can host nanoparticles and immune cues to stage delivery—an early antibacterial push followed by pro-resolution signals—using thermo/enzymatic or external-stimuli triggers (Sikder et al., 2021; Nazir et al., 2022; Mu et al., 2025). Accordingly, hydrogels are best positioned for localized, defect-centric immunomodulation, complemented by systemic drugs for syndromic inflammation and by targeted nanoparticles for specificity.

Despite these challenges, the prospects for clinical translation are quite promising. The medical need is clear: chronic bone infections and large bone defects remain very difficult to manage, and failure often means amputation or life-long disability. Immunomodulatory hydrogels could significantly improve outcomes by reducing the recurrence of infection and enhancing the speed and quality of bone repair. Regulatory agencies are also increasingly familiar with combination biomaterial products (for example, bone morphogenetic protein-2 delivered on a collagen scaffold is FDA-approved for certain bone fusion applications). As more preclinical data accumulates demonstrating safety and efficacy–including studies in large animal models that closely mirror human clinical scenarios–the path to first-in-human trials becomes shorter. A potential early milestone might be a compassionate use or small pilot trial in patients who have no good alternatives (for example, a patient with a massive infected bone defect not responsive to standard care). A successful outcome in such a case could galvanize the field and attract broader clinical and commercial interest.

Furthermore, these hydrogels fit well into the paradigm of personalized medicine. In the future, a surgeon might take a small sample of a patient’s blood or inflammatory tissue, have it analyzed for key immune markers, and then select a hydrogel formulation best suited for that patient’s immune profile. Alternatively, a modular hydrogel “kit” could be available, where the surgeon mixes an “antibiotic module” and an “immunomodulator module” of choice into the base hydrogel, based on the specific bacteria involved and the patient’s immune status. The foundation laid by current research indicates that immunomodulatory hydrogels can indeed be rationally designed and tuned to meet such tailored needs.

## 7 Conclusion

The treatment of infection-associated bone defects represents one of the most challenging problems in orthopedic medicine, requiring a paradigm shift from traditional antimicrobial approaches to sophisticated bioengineering solutions that address both infection control and tissue regeneration. This review demonstrates that immunomodulatory hydrogels offer a transformative therapeutic strategy by targeting the fundamental cellular mechanisms underlying failed bone healing in infected environments. By concurrently eliminating pathogens and modulating the immune response to support healing, these hydrogels bridge the gap between infection management and regenerative medicine. Continued research and successful translation of these approaches may pave the way for improved outcomes in patients suffering from chronic bone infections and large bone defects, where current treatments are often insufficient.
